# Establishment of a Pediatric COVID-19 Biorepository: Unique Considerations and Opportunities for Studying the Impact of the COVID-19 Pandemic on Children

**DOI:** 10.21203/rs.3.rs-42030/v1

**Published:** 2020-08-10

**Authors:** Rosiane Lima, Elizabeth Gootkind, Denis De La Flor, Laura Yockey, Evan Bordt, Paolo D’Avino, Shen Ning, Katerina Heath, Katherine Harding, Jaclyn Zois, Grace Park, Margot Hardcastle, Kathleen A. Grinke, Sheila Grimmel, Pamela J. Forde, Susan P. Davidson, Kathryn E. Hall, Anne Neilan, Juan D. Matute, Paul H. Lerou, Alessio Fasano, Jessica E. Shui, Andrea G. Edlow, Lael M. Yonker

**Affiliations:** Massachusetts General Hospital; Massachusetts General Hospital; Massachusetts General Hospital; Massachusetts General Hospital; Massachusetts General Hospital; Massachusetts General Hospital; Massachusetts General Hospital; Massachusetts General Hospital; Massachusetts General Hospital; Massachusetts General Hospital; Massachusetts General Hospital; Massachusetts General Hospital; Massachusetts General Hospital; Massachusetts General Hospital; Massachusetts General Hospital; Massachusetts General Hospital; Massachusetts General Hospital; Massachusetts General Hospital; Massachusetts General Hospital; Massachusetts General Hospital; Massachusetts General Hospital; Massachusetts General Hospital; Massachusetts General Hospital; Massachusetts General Hospital

**Keywords:** COVID-19, SARS-CoV-2, Multisystem Inflammatory Syndrome in Children (MIS-C), Viral transmission, Viral susceptibility, Biorepository, Biobank, Pediatric

## Abstract

**Background:**

COVID-19, the disease caused by the highly infectious and transmissible coronavirus SARS-CoV-2, has quickly become a morbid global pandemic. Although the impact of SARS-CoV-2 infection in children is less clinically apparent, collecting high-quality biospecimens from infants, children and adolescents in a standardized manner during the COVID-19 pandemic is essential to establish a biologic understanding of the disease in the pediatric population. This biorepository enables pediatric centers world-wide to collect samples in a standardized manner to drive forward our understanding of COVID-19 by addressing specific pediatric and neonatal COVID-19-related questions.

**Methods:**

A broad study was implemented with strategic enrollment guidelines to include patients seen in urgent care clinics and hospital settings, neonates born to SARS-CoV-2 infected mothers, and asymptomatic children. The methodology described here, details the importance of establishing collaborations between the clinical and research teams to harmonize protocols for patient recruitment and sample collection, processing and storage.

**Results:**

Considerations and challenges facing enrollment of neonatal and pediatric cohorts are described. A roadmap is laid out for successful collection, processing, storage and database management of multiple pediatric samples such as blood, nasopharyngeal and oropharyngeal swabs, sputum, saliva, tracheal aspirates, stool, and urine. Using this methodology, we enrolled 327 participants, who provided a total of 972 biospecimens.

**Conclusions:**

Pediatric biospecimens will be key in answering questions relating to viral transmission by children, differences between pediatric and adult viral susceptibility, and, immune responses, the impact of maternal SARS-CoV-2 infection on fetal development, and factors driving the Multisystem Inflammatory Syndrome in Children. The specimens in this biorepository will allow necessary comparative studies between children and adults, help determine the accuracy of current pediatric viral testing techniques, in addition to, understanding neonatal exposure to SARS-CoV-2 infection and disease abnormalities. The successful establishment of a pediatric biorepository is critical to provide insight into disease pathogenesis, and subsequently, develop future treatment and vaccination strategies.

## Background:

The global pandemic of COVID-19, caused by the highly infectious and transmissible coronavirus, SARS-CoV-2, has become a leading cause of death in older adults[1]. While adults can develop life-threatening complications such as pneumonia, acute respiratory distress syndrome (ARDS), and sepsis from SARS-CoV-2 infection, its impact on children is less clinically apparent and needs to be studied. Collecting high-quality biospecimens from infants, children and adolescents in a standardized manner during the COVID-19 pandemic is essential for understanding the biologic consequences of SARS-CoV-2 infection in children.

Specific questions that must be addressed revolve around the role children play in viral transmission, differences in pediatric viral susceptibility and immune responses, which could guide potential therapies for adults, the impact of maternal SARS-CoV-2 infection on fetal development, and factors driving the development of severe hyperinflammatory shock and cardiac damage seen in Multisystem Inflammatory Syndrome in Children (MIS-C). Outlined here is a roadmap for establishing a biorepository of specimens obtained from infants, children and adolescents during the COVID-19 pandemic. Special attention is provided to pediatric-specific considerations in the establishment of a biorepository during the COVID-19 pandemic. The goal is to enable pediatric centers world-wide to collect samples in a standardized manner to drive forward our understanding of COVID-19.

## Methods:

The impact of SARS-CoV-2 infection on infants and children is not well-defined. Children are typically asymptomatic or mildly symptomatic, although some can develop significant complications requiring intensive care. In order to capture the full range of SARS-CoV-2 infection in the pediatric population, a broad study design was implemented, including patients seen in urgent care clinics and hospital settings, neonates born to SARS-CoV-2-infected mothers, and asymptomatic children. Each study population required specific tailoring of study conduct to effectively and efficiently collect critical samples.

To establish a COVID-19 biorepository before the surge of cases, protocols were rapidly submitted to our Institutional Review Board (IRB, MGH IRB#2020P000955) and the Institutional Biosafety Committee (IBC, MGH IBC#2020B000061) for approval. The entire research team was properly trained on Biosafety Level 2-Plus (BSL2+) procedures, as required for handling SARS-CoV-2 specimens. Close collaborations with adult biorepositories within the hospital ensured consistency, and, harmonization in biospecimen collection and processing across patient cohorts, facilitating high-quality comparisons between patient groups. A coordinated effort by clinical research coordinators, laboratory technicians, graduate students, and postdoctoral fellows was essential for enrolling patients, coordinating sample collection with clinical teams, maintaining IRB approval, and for processing and storing high-quality biospecimens. Central to the operation, physician-scientists in Pediatrics, Neonatology, Medicine-Pediatrics, and Obstetrics-Gynecology collaborated to harmonize sample collection protocols, establish clinical connections, and provide clinical and scientific context to COVID-19-related research in the neonatal and pediatric population. Open communication, frequent quality checks, and accessible leadership was paramount, in addition to coordination with patients’ clinical care teams. Samples were collected in the clinical setting by the clinical team members to accommodate COVID-19 infection control guidelines and limit personal protective equipment (PPE) use. Cornerstones of the biorepository included open dialogue between research and clinical team members, a sensitivity to procedures required for specimen collection in children, and clear documentation of study participation and sample collection.

### Patient enrollment and sample collection:

**Figure 1:**
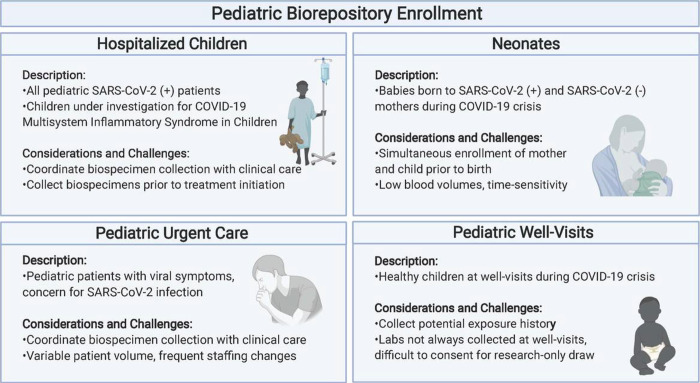
Schematic of the recruitment strategies used to pursue the collection of pediatric samples for the Pediatric COVID-19 biorepository (Created with BioRender.com).

In order to include pediatric and neonatal patients from a range of clinical presentations for COVID-19, we established 4 cohorts of patients: **1)** Pediatric patients with mild-moderate COVID-19, presenting to the MGH COVID-19 urgent care clinics, **2)** Pediatric patients with severe COVID-19 or MIS-C requiring hospitalization, **3)** Newborns born to mothers infected with SARS-CoV-2 at any point during their pregnancy, and, infants born to non-infected mothers, and **4)** asymptomatic children at well-visits with possible SARS-CoV-2 exposure. Figure 1 provides a schematic of the recruitment strategy. Potential participants in all cohorts were contacted via telephone to verbally review the informed consent. Participants or their parent/guardian selected which biospecimens they would provide. One copy of the signed consent form was emailed to enrolled patients, and another was uploaded into the electronic medical record flagging the patient as a research participant, clearly documenting research participation for the clinical care teams. Participants were assigned a unique study ID number using REDCap, a secure, centralized online database platform that allows simultaneous recruitment at multiple sites without risking assigning the same number to multiple patients. The clinical team obtained biospecimens to minimize risk of SARS-CoV-2 transmission and limit PPE use.

#### 1) Pediatric patients with mild-moderate COVID-19

As most children did not require hospital-level care, significant efforts were made to enroll patients in the outpatient setting. COVID-19 screening clinics, called Respiratory Infection Control clinics, were established at Massachusetts General Hospital. As COVID-19 symptoms are non-specific and current diagnostic reporting is time-delayed, all pediatric patients presenting to the COVID-19 screening clinics were eligible to participate in the biorepository.

In Respiratory Infection Control clinics, participants who signed informed consent could provide nasopharyngeal, oropharyngeal swabs and/or blood. Stool and urine were not collected given time limitations of clinic visits and patient flow patterns established to minimize potential COVID-19 exposures to clinical staff. Blood was collected into one tube with an EDTA anticoagulant (EDTA tube) (BD), one serum separator tube (SST) (BD), and a PAXgene RNA tube (BD). Blood volumes varied, depending on the age and weight of the patient, in accordance with limits established by the IRB. The aerosolizing procedure of collecting nasopharyngeal and oropharyngeal swabs into 15 mL falcon tubes, containing 3 mL phosphate buffered saline (PBS) (Gibco), was performed by clinical team members wearing N95 mask, face shield, protective outer gown and disposable gloves.

#### 2) Patients with severe COVID-19 or Multisystem Inflammatory Syndrome in Children requiring hospitalization

Pediatric patients who were hospitalized with suspicion of SARS-CoV-2 exposure and/or symptoms concerning for SARS-CoV-2 infection or MIS-C were approached. Presenting symptoms included fever, rash, dyspnea, cough, nausea, vomiting or diarrhea. Patients were approached by the clinical team and upon declaring interest, a member of the research staff contacted the patient and family to obtain informed consent. Hospitalized patients could opt to provide urine, stool, sputum, or if intubated, tracheal aspirates, in addition to blood, nasopharyngeal and oropharyngeal swabs. Planned sample collection was discussed with the clinical team. Phlebotomy was aligned with clinical blood draws, when feasible, although participants had an option to undergo a separate venipuncture for research purposes. Blood was collected into an EDTA tube, an SST tube, and a PAXgene RNA tube. Repeat samples were collected on alternating days, as feasible. Based on daily coordination between the research and clinical teams, discarded blood from clinical labs were also obtained from hospitalized patients.

#### 3) Newborns born to mothers with and without SARS-CoV-2 infection

Pregnant women with confirmed SARS-CoV-2 infection followed in the MGH obstetrics practice, presenting to the Labor and Delivery (L&D) Unit, or hospitalized for SARS-CoV-2 illness, were approached to enroll their infant in the Pediatric COVID-19 Biorepository following birth. When universal screening for SARS-CoV-2 infection was initiated on all pregnant women admitted to L&D, asymptomatic SARS-CoV-2 positive patients were identified and offered enrollment. Women who tested negative for SARS-CoV-2 were also approached as a control group. The pregnant mothers were simultaneously offered enrollment in the companion Obstetric COVID-19 Biorepository, which included collection of placental biopsies, umbilical cord blood, and other maternal samples. The clinical team assessed the patient’s interest in the Biorepository, then a member of the research staff contacted the patient to obtain informed consent. Parents could opt to have newborn blood, nasopharyngeal and oropharyngeal swabs, urine, stool, and (if intubated) tracheal aspirates collected. Blood was collected via heel stick between 24–36 hours of life, simultaneously with the heel stick for clinical newborn screening, into two EDTA microtainer tubes (BD). Research nasopharyngeal and/or oropharyngeal swabs were obtained after 24 hours of life, batched at the time of the nasopharyngeal swab for SARS-CoV-2 testing, if performed clinically. Stool and urine were collected on day of life 0 and 2. Stool was collected directly from the diaper. Urine was collected by placing cotton balls in the diaper, then transferring the urine-soaked cotton balls into a specimen cup for transport. If the recruited newborn was intubated for clinical indications, tracheal aspirates were collected at the time of clinical suctioning.

#### 4) Asymptomatic children with potential SARS-CoV-2 exposure

Children presenting for their 2-, 3-, or 4-year annual well-child visit with their pediatrician for planned phlebotomy were eligible to participate in this cohort. Eligible patients were identified by study staff and clinicians. If appropriate, researchers contacted the parents via telephone prior to their visit to explain the research and obtain informed consent. Blood and saliva were collected during their clinical phlebotomy. Saliva collection is not considered an aerosolizing procedure; thus, these specimens could be collected in clinic without the need for N95 mask use. The specimens were immediately transported to the laboratory for processing.

### Data collection:

REDCap databases were used to record all study data, including: 1) An enrollment log serving as the decoding log. Study ID numbers were assigned consecutively across all four patient groups; 2) A laboratory processing database with pertinent processing and freezer storage location information; 3) A chart review database, with demographic and clinical data, including COVID-19 exposures, SARS-CoV-2 polymerase chain reaction (PCR) results, symptoms, and outcomes; 4) A question-response database about COVID-19 exposures and risk factors, specifically for the well-visit cohort.

### Specimen Transport and Processing:

In accordance with specimen transport guidelines, specimens were sealed in a leak-proof container labeled with subject’s study ID, then placed in a tight-sealed, biohazard-labeled, secondary container with a rigid outer container and lockable lid (e.g. Igloo cooler) for transport to the laboratory. Blood samples were processed following BSL2 safety guidelines, with a lab coat, nitrile/latex gloves, and a face shield or safety goggles. All other samples, including nasopharyngeal and oropharyngeal swabs, sputum, saliva, tracheal aspirates, stool, and urine were processed following BSL2 + safety guidelines. BSL2 + safety precautions require all samples to be processed in a certified biosafety cabinet (BSC), class II A2, with intake airflow. Well-trained laboratory personnel handling infectious specimens were required to wear closed-front water impermeable gowns, double nitrile/latex gloves, sleeve covers, and a face shield. Outer gloves were removed when moving away from the BSC and replaced with a new glove when returning to work in the BSC.

#### Plasma

Blood samples collected in tubes with an EDTA anticoagulant were stored at room temperature until processed, within 24 hours of collection. Tubes were spun at 1000 g for 10 minutes with brake activated. Plasma was then collected, aliquoted, stored at −80 °C, and logged in the REDCap database.

#### Peripheral blood mononuclear cells (PBMCs)

**Figure 2:**
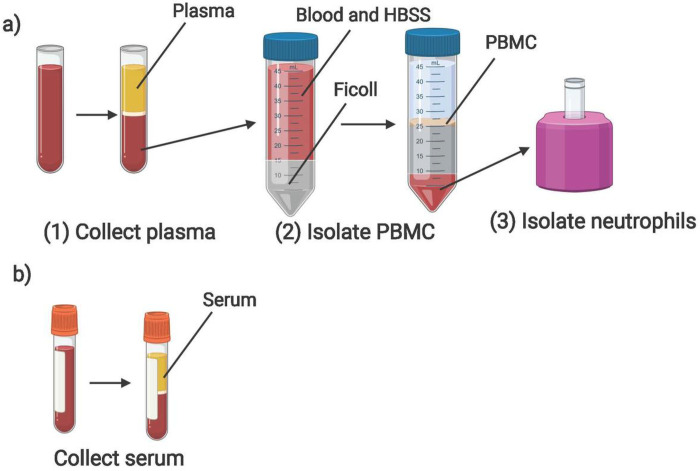
Overview of laboratory blood processing procedures following BSL2 containment guidelines depicting steps for a) collection of plasma, isolation of PBMC and PMN, from blood collected into an EDTA tube, and, for b) collection of serum from an SST blood tube (Created with BioRender.com).

Immediately following the removal of plasma, samples with greater than 2 mL initial volume were processed for PBMC isolation using a Ficoll density gradient[2]. Briefly, blood was transferred into a 50 mL conical tube, then diluted 1:1 with Hanks’ Balanced Salt Solution without calcium or magnesium (HBSS minus) (Gibco). This diluted blood was then gently layered on top of Ficoll-Paque Plus (GE Healthcare) at 2:1 ratio (2 volumes of blood diluted with HBSS minus to 1 volume Ficoll). Careful attention was made to avoid any mixing of blood with the Ficoll layer. The conical tube was then centrifuged at 1000 g for 30 minutes at room temperature with brake inactivated to allow adequate layering of cellular components. The cloudy ring below the plasma and above the Ficoll (i.e. the PBMC layer) was collected and transferred to a new 15 mL conical tube, with HBSS minus added to bring the volume to 15 mL (Fig. 2a). This tube was then centrifuged at 330 g for 10 minutes, with high brake activated. The supernatant was removed, the PBMC pellet was again washed with HBSS minus, and then resuspended in 10 mL HBSS minus for counting. Cell count was obtained by diluting 10 μL of sample with 90 μL of trypan blue, mixed, and sampled on a hemocytometer. Cells were then frozen in freshly-prepared freezing medium (RPMI 1640 Medium with 1% penicillin-streptomycin, L-glutamine, 1% sodium pyruvate, 1% non-essential amino-acids, and 20% Fetal Bovine Serum (FBS) (Sigma)) with 10% DMSO (Sigma) for a goal concentration range of 5–10 million cells/vial, placed in a chilled Mr. Frosty filled with isopropanol, then immediately placed at −80 °C. Final concentration (5–10 million cells per 1 mL of freezing medium) and number of aliquot vials were logged. The following day, PBMC cryovials were moved to a liquid nitrogen freezer for long term storage, and location was recorded in specimen log.

PBMCs were isolated within 24 hours of phlebotomy, although higher cell counts were obtained if isolated within 3–4 hours of collection. If less than 5 mL blood was collected, a 15 mL conical tube, rather than a 50 mL conical tube could be used for Ficoll layering. Fresh freezing media were made throughout the day for each sample batch.

#### Neutrophils

Neutrophils were extracted from the red blood cell layer that remained following the collection of PBMCs (Fig. 2a). Neutrophils were isolated using EasySep Direct Human Neutrophil Isolation Kit (StemCell Technologies). The remaining blood layer was incubated with EasySep Direct RapidSpheres and EasySep Direct Human Neutrophil Isolation Cocktail, then diluted in EasySep Buffer. Neutrophils were isolated by successive negative magnet selection using EasySep magnets, then counted using a hemocytometer and aliquoted into Eppendorf tubes for RNA extraction (1 × 10^5^ cells/tube) or DNA analysis (5 × 10^6^ cells/tube). Neutrophils designated for RNA extraction were resuspended in 100 μL of RNA lysis buffer (TCL) (Qiagen) with 1% β-mercaptoethanol (Sigma), immediately stored at −80 °C and logged. Neutrophils planned for DNA analysis were pelleted then directly stored at −80 °C and logged.

For RNA extraction steps, a cleaning agent, such as RNaseZAP should be used to remove RNAse from the working surface. RNA lysis buffer should be newly made for each sample using a 10:1 TCL to β-mercaptoethanol ratio.

#### Serum

Serum samples were collected from blood drawn into serum separator tubes without any anticoagulant (BD). Blood was kept at room temperature, standing upright for 30–60 minutes, then spun at 1200 g for 10 minutes with brake activated. Serum was then collected, aliquoted, stored, and logged (Fig. 2b).

#### Nasopharyngeal and Oropharyngeal Swabs

**Figure 3:**
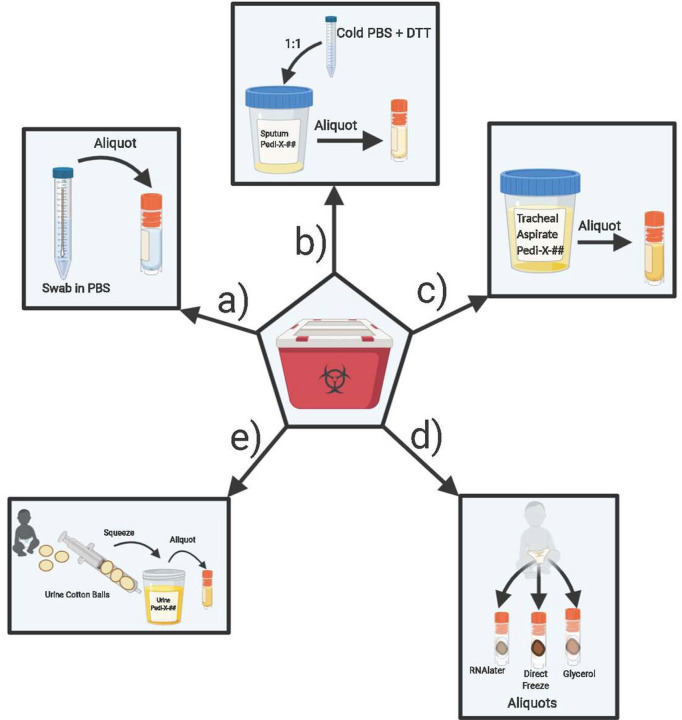
Overview of laboratory processing procedures completed following BSL2+ containment guidelines include: a) aliquoting nasopharyngeal swabs and oropharyngeal swabs, b) aliquoting sputum/saliva samples, c) aliquoting tracheal aspirates d) aliquoting stool samples and e) aliquoting urine from urine cotton balls collected from patient’s diaper, (Created with BioRender.com).

Swab samples were delivered in phosphate buffered saline (PBS)[3]. Samples were directly aliquoted into 1 mL aliquots, then immediately stored at −80 °C and logged (Fig. 3a).

#### Sputum/Saliva

Samples collected into a collection cup were mixed well at 1:1 ratio with 500 mM DL-Dithiothreitol (DTT) (Sigma)/PBS solution according to CDC recommendations. Diluted samples were then divided into 1 mL aliquots, volume permitting, immediately stored at −80 °C and logged (Fig. 3b).

#### Tracheal aspirates

Aspirates collected into a sterile collection cup were divided into 1 mL aliquots (1 mL/vial), immediately stored at −80 °C and logged (Fig. 3c).

#### Stool

Stool samples collected from a diaper or specimen cup were divided using a micro spatula volume permitting, into cryovials with 1 mL RNAlater (Invitrogen), empty cryovials without any additive/reagent, up to the 1.5 mL tube mark, and cryovials with 1 mL Buffered Glycerol Saline (Fisher). Stool samples were fully submerged in RNAlater or glycerol solution prior to immediate storage at − 80 °C. Samples were logged onto database (Fig. 3d).

#### Urine

Urine samples collected with cotton balls placed inside baby diapers were transferred using forceps, to a 10 mL syringe to dispense at most 1 mL of fluid into cryovials and immediately stored at − 80 °C. Samples collected into a tube or a sterile collection cup were aliquoted into cryovials (at 1 mL at most/vial) and immediately stored at − 80 °C (Fig. 3e).

Supplies required for specimen collection and processing are listed in Supplemental Table 1.

## Results:

The Pediatric COVID-19 biorepository enrolled 327 pediatric and neonatal patients from a range of clinical presentations, including 178 patients from the urgent care/Respiratory Infection Control clinic, 48 hospitalized children, 85 newborns born to mothers with or without SARS-CoV-2 infection, and 16 asymptomatic children presenting for their well-visits. The average age was 11 (±8) years for enrolled children and adolescents and 1.3 (±1.3) days for newborns. Equal gender distribution was seen, except more males enrolled in the hospitalized cohort (60%, n = 29). Sixty-four participants were positive for SARS-CoV-2 by clinical testing, most of whom were seen in the Respiratory Infection Control clinic, while 21 patients were diagnosed with MIS-C, all of whom were hospitalized. The one patient presenting to the Respiratory Infection Control clinic with MIS-C was ultimately hospitalized. Table 1 characterizes total enrollment number, age, gender, SARS-CoV-2 infection status and MIS-C diagnosis within each enrollment site.

A total of 972 biospecimens were collected. These biospecimens included 295 blood samples, 181 nasopharyngeal swabs, 145 nasopharyngeal swabs, 172 stool samples, 154 urine samples, 4 tracheal aspirate samples, and 21 sputum/saliva samples. Hospitalized patients and newborns had the option of providing subsequent sampling. Table 2 depicts the sample collection from each enrollment site.

## Discussion:

The goal of the biorepository is to provide high quality biospecimens for studies understanding how infants and children are impacted by and contribute to COVID-19. Establishing a standardized biorepository collection protocol facilitates comparison of samples across institutions and with adult biorepositories. Key neonatal and childhood factors of interest that will be studied using this biorepository focus on: 1) informing pediatric contribution to viral transmission, 2) teasing apart the dichotomy between pediatric and adult immune responses to COVID-19, 3) ascertaining the impact of maternal SARS-CoV-2 infection on child fetal development, and 4) elucidating factors driving the MIS-C.

### Inform pediatric contribution to viral transmission

In this study, nasopharyngeal and oropharyngeal swabs were collected from pediatric patients presenting with symptoms concerning for SARS-CoV-2 infection in both the outpatient and hospitalized setting, from newborns born to mothers infected with SARS-CoV-2, and from healthy controls. Additionally, saliva was collected from young children presenting for their annual well-visit. Blood, tracheal aspirates, stool, and urine were also collected from the hospitalized patients and newborns for assessment of viral load. Questions relating to the role of viral carriage in the pediatric population can be addressed using these samples. Case reports suggest that asymptomatic children may carry high viral loads despite lack of symptoms[4, 5]. In adults, severe infection is not necessarily associated with a significant increase in viral loads by nasopharyngeal swab[6], and, asymptomatic individuals appear to have equal viral loads as symptomatic individuals[7]. The potential correlations between viral load, symptoms, and exposures have yet to be clarified in the pediatric population. Age-stratification within adults show no differences in viral load across age-groups, although younger patients were less likely to develop severe disease[6], thus a similar comparison among children would be informative. Additional questions remain as to whether there are risk factors affecting viral load density in children, including household contacts or other environmental factors. Viral studies are also needed to determine accuracy of viral testing techniques within the pediatric population. Including infants born to COVID-19 infected mothers will allow assessment of viral exposure in the airway, and through the meconium, giving important insight into neonatal exposure to SARS-CoV-2 infection. Understanding how children are infected with SARS-CoV-2 will provide critical insight into how viral loads may impact disease severity in children, and how children may contribute to viral transmissibility driving this pandemic. These data will be critical to making decisions about risk factors for re-opening of schools and childcare as the pandemic progresses.

### Tease apart the dichotomy between pediatric and adult immune responses to COVID-19

COVID-19 results in a major apparent dichotomy of immune response between children and adults. Children often develop mild infections whereas adults more commonly develop severe disease associated with high levels of mortality[8]. Neonates appear to be unaffected, even when born to COVID-19 positive mothers[9]. It has been postulated that children are less impacted by viral infection because they have fewer angiotensin-converting enzyme 2 (ACE2) viral binding sites[10], although the research thus far remains conflicted. In this study, RNA obtained from nasopharyngeal and oropharyngeal swabs, and/or saliva collected from neonates, children, and young adults, can be used to characterize ACE2 expression, and potentially shed light on the availability of viral binding sites across the age span. Further, prior research has shown immunosenescence in aged individuals, which affects T cell and B cell function, and cytokine production by innate immune cells[11]. It is yet to be evaluated as to whether this plays a central role in the age differences in the morbidity and mortality from COVID-19. Additionally, MIS-C is shown to be driven by a cytokine storm and macrophage activation[12]. Peripheral blood monocytes, plasma, serum, and neutrophil RNA collected as part of this biorepository can be used to answer these questions. Understanding the disease abnormalities may provide key insight into therapeutic targets. This study collects plasma, serum, PBMCs, and neutrophil RNA from SARS-CoV-2 infected and uninfected children with a range of symptoms for comparison.

### Ascertain the impact of maternal SARS-CoV-2 infection on fetal development

Neonatal development intimately depends on maternal health. Prior infections and disease states causing maternal inflammatory activation and cytokine storm have resulted in increased risk of autism spectrum disorder, schizophrenia, cerebral palsy, cognitive delay, depression, and bipolar disorder in exposed children [13–15]. The effect of the SARS-CoV-2 hyperinflammatory milieu on the developing fetus is yet to be seen. This biorepository, partnered with the Obstetric COVID-19 Biorepository will obtain placental tissue, cord blood, maternal and neonatal biospecimens to address these critical questions.

### Elucidate factors driving the Multisystem Inflammatory Syndrome in Children (MIS-C)

Following a mild or symptomatic infection of COVID-19, children can develop a severe, post-infectious inflammatory response syndrome, termed Multisystem Inflammatory Syndrome in Children (MIS-C), which is characterized by hyperinflammatory shock[16], “Kawasaki-like” cardiac damage, and possible death[17]. Risk factors for developing MIS-C and biomarkers predicting severe complications need to be identified. These specimens collected through this Pediatric COVID-19 biorepository will be used to characterize the immune responses driving MIS-C in hopes of mitigating this life-threatening complication.

## Conclusion:

Although children were initially felt to be spared from COVID-19, it has become clear that much needs to be learned as to how children and newborns are impacted by the pandemic. Research is needed to address viral transmission by children, differences in pediatric viral susceptibility and immune responses, the impact of maternal SARS-CoV-2 infection on fetal development, and factors driving the MIS-C. This Pediatric COVID-19 Biorepository will serve as an important resource providing critical insight into disease pathogenesis, COVID-19-susceptibility, and future treatment and vaccination strategies.

## Supplementary Material

Supplement

## Figures and Tables

**Table 1:** Characteristics of enrolled patients.

Total enrolled (N=327)	Urgent Care (n=178)	Hospitalized (n=48)	Newborn (n=85)	Well visit (n=16)
Age, average (SD)	12.3 years (7.9)	8.5 years (8.2)	1.3 days (1.3)	4.0 years (4.8)
*%* Male (number)	50(89)	60(29)	51(43)	50(8)
SARS-CoV-2 per (+)	46	18	0	0
MIS-C	1	20	0	0

Age, sex, SARS-CoV-2 clinical testing results, and MIS-C status are described. Polymerase chain reaction of nasopharyngeal swab was clinically used to determine SARS-CoV-2 infection status.

**Table 2:** Biospecimens collected from each patient cohort.

Total number of samples (N=972)	Blood (n=295)	Oropharyngeal swab (n=181)	Nasopharyngeal swab (n=145)	Stool (n=172)	Urine (n=154)	Tracheal aspirate (n=4)	Sputum/saliva (n=21)
Urgent Care	86	105	79	0	3	0	7
Hospitalized	147	43	44	46	58	3	10
Newborn	55	29	19	126	93	1	0
W ell visit	7	4	3	0	0	0	4

Enrolled subjects had the option of providing all, some, or no biospecimens. Repeat biospecimen collection could occur if participants re-presented to care, or if hospitalized for multiple consecutive days.

## Abbreviations

ACE2: Angiotensin-converting Enzyme 2
ARDS: Acute Respiratory Distress Syndrome
BSC: Biosafety Cabinet
BSL(2+): Biosafety Level (2 plus)
EDTA: Ethylenediamine Tetraacetic Acid
FBS: Fetal Bovine Serum
HBSS: Hank’s Balanced Salt Solution
IRB: Institutional Review Board
IBC: Institutional Biosafety Committee
L&D: Labor and Delivery
MGH: Massachusetts General Hospital
MIS-C: Multisystem Inflammatory Syndrome in Children
mL: Milliliters (unit of measurement)
PCR: Polymerase Chain Reaction
PMBC: Peripheral Mononuclear Blood Cells
PBS: Phosphate Buffered Solution
PPE: Personal Protective Equipment
RNA: Ribonucleic Acid
