# Supplementary material for: Establishment of a Pediatric COVID-19 Biorepository: Unique Considerations and Opportunities for Studying the Impact of the COVID-19 Pandemic on Children
Source: Res Sq. 2020 Aug 10:rs.3.rs-42030. Preprint. [Version 1] doi: 10.21203/rs.3.rs-42030/v1 (PMC7430592; doi:10.21203/rs.3.rs-42030/v1)
Supplement: Supplementary file 1 [file SupplementalTable1.docx]

**Supplemental Table 1**: List of supplies required for specimen collection, processing, and storage in the Pediatric COVID-19 Biorepository. 

| **Company - Catalog No.** | **Item Description** |
| --- | --- |
| **Pediatric Sample Collection Kit Supplies** |  |
| BD 762165 | PAXgene Blood RNA tubes (2.5mL) |
| BD 367863 | 6mL K2 EDTA tubes |
| BD 367841 | 2mL K2  EDTA tubes |
| BD 366643 | 10mL K2 EDTA tubes |
| BD 367983 | 3.5mL SST tubes |
| BD 365967 | SST Microtainers |
| BD 365974 | EDTA Microtainers |
| BD 367671 | 2mL Sodium Heparin tubes |
| Copan 552C | Oropharyngeal Flocked Swab - 80mm break point in dry tube |
| Fisher 23600956 (Copan 518CS01) | Nylon Flocked Dry Swabs (nose swabs) |
| SPC400 | MediChoice specimen container (4oz/120 cc) |
| Om Main Street (SB0609) | Biohazard specimen bag- ziplock w. outside pocket |
| Lab Loc Specimen Bags | Clear Biohazard Specimen Transport Bags |
| Staples 479880 | 1x 2 5/8 Label Sheets for patient kits |
| **Personal Protective Equipment** |  |
| Fisher 19-181-600A | Face Shields |
| Precept 5030 | Disposable sterile closed-front impermeable gowns |
| Fisher 19-830-003 | Sterile Sleeve Covers |
| Fisher 19-041-172D / Genesee 44-106L | Large Nitrile Exam Gloves, Green |
| Fisher 19-041-172C / Genesee 44-106M | Medium Nitrile Exam Gloves, Green |
| Fisher 19-041-172B / Genesee 44-106S | Small Nitrile Exam Gloves, Green |
| Genesee 44-100L | X-GEN Nitrile Examination Gloves, L Cobalt Violet Blue |
| Genesee 44-100M | X-GEN Nitrile Examination Gloves, M Cobalt Violet Blue |
| Genesee 44-100S | X-GEN Nitrile Examination Gloves, S Cobalt Violet Blue |
| **COVID-19 Biospecimen Processing Supplies** |  |
| Gibco 10010049 | PBS (1X) |
| Sigma D0632-1G | DL-dithiothreitol powder |
| Thermo Sci R21650 | Buffered glycerol saline |
| Invitrogen AM7021 | RNA Later Solution |
| GE Healthcare    17-1440-03 | Ficoll-Paque Plus |
| BP2311/Sigma D8418 | DMSO (dimethyl sulfoxide) |
| F4135 | Fetal Bovine Serum (FBS) |
| Sigma R7638 - 6x500ML | RPMI 1640 Dutch Modification |
| Gibco 14175095 | HBSS (-) |
| 15140-122 | Pen/Strep (100mL) |
| Sigma M6250-10mL | β-Mercaptoethanol (10mL) |
| USA Scientific 9187-1700 | Cryolabels sheets |
| Fisher Scientific AM12645 | 20µL barrier tips |
| Genesee 24-430 | 1000µL barrier tips |
| Genesee 24-412 | 200µL barrier tips |
| Thermo 5000-0020 | Nalgene 2.0mL Cryovials |
| USA Scientific 1615-5510 | 1.5mL Eppendorf tubes |
| Corning 352097 (Fisher 1495970C) | 15mL Conical tube |
| Corning 430052 | 15mL conical tubes |
| Corning 430829 | 50mL Conical Tubes |
| Corning 352054 | 5mL Polystyrene round bottom tube |
| Fisher SCGP00525 | Steriflip – vaccum filter for 50mL falcon |
| Olympus plastic 12-106 | 25mL Serological Pipette |
| Costar 4488 | 10mL Serological Pipette |
| Costar 4487 | 5mL Serological Pipette |
| BD 309604 | 10mL Syringes |
| Kimtech 341133 | Large Kimwipes |
| Kimtech 34155 | Small Kimwipes |
| VWR 95042-566 | Chemical resistant marker |
| Genesee 21-121W | Cryogenic Storage Boxes |
| Decon 2701 | Ethanol 200 proof |
| **Neutrophil Isolation** |  |
| Stem Cell Technologies Catalog # 20144 | EasySep Buffer – Cell separation buffer |
| Stem Cell Technologies Catalog # 19666 | EasySep Direct Human Neutrophil Isolation Kit |
| Stem Cell Technologies Catalog # 18000 | EasySep Magnet (for up to 2mL whole blood) uses standard 12 x 75 mm (5mL) polystyrene tube |
| Stem Cell Technologies Catalog # 18002 | Easy 50 EasySep Magnet (5 to 25mL whole blood) - uses standard 50mL conical tubes |
| Qiagen Catalog # 1031576 | Buffer TCL |
| **Miscellaneous** |  |
| Grainger 1HUG6 (Fisher NC0484599) | Igloo container |
